# Case Report: Pulsed electric field treatment of metastatic liver disease demonstrating a possible abscopal response

**DOI:** 10.3389/fimmu.2026.1592656

**Published:** 2026-03-19

**Authors:** Sandra Gad, Michael Mohnasky, Zachary Schrank, Jonathan David Sorah, Jacob Myhre, Bryan Harris, Hui Wang, Bahareh Gholami, Ali Afrasiabi, Nima Kokabi

**Affiliations:** 1 Division of Vascular and Interventional Radiology, Department of Radiology, University of North Carolina at Chapel Hill, Chapel Hill, NC, United States; 2 St. George’s University, Chapel Hill, Grenada; 3 School of Medicine, University of North Carolina at Chapel Hill, Chapel Hill, NC, United States; 4 Division of Oncology, Department of Medicine University of North Carolina at Chapel Hill, Chapel Hill, NC, United States; 5 Department of Pathology and Laboratory Medicine, University of North Carolina at Chapel Hill, Chapel Hill, NC, United States

**Keywords:** AblaHon, abscopal, adrenocorHcal carcinoma, checkpoint inhibitors, liver tumor, pulse electric field

## Abstract

Adrenocortical carcinoma (ACC) is a rare and aggressive endocrine malignancy, typically presenting at an advanced stage with distant metastases. We report a unique case of a 49-year-old female with metastatic ACC in the liver who exhibited an abscopal effect following pulse electric field (PEF) ablation. The patient initially presented with hypertension, male-patterned hair loss, voice hoarseness, and weight gain and was subsequently diagnosed with a large left adrenal mass along with liver and pulmonary metastases. Following surgical interventions—including left salpingo-oophorectomy, hepatic wedge resection, and left adrenalectomy—and initial systemic treatment with doxorubicin, etoposide, and cisplatin-mitotane (EDP-M), disease progression prompted initiating therapy with pembrolizumab and mitotane. The patient then received Y90 treatment in the right lobe; however, imaging demonstrated viable metastatic disease in the left lobe, which prompted PEF treatment. The patient received PEF almost 7 months post-pembrolizumab and mitotane initiation. Remarkably, two months post-PEF treatment, PET/CT imaging demonstrated complete resolution of three of the four untreated lesions, suggesting an abscopal response. This case adds to the limited literature on PEF ablation, highlighting its potential not only as a local control strategy but also as an adjunct to systemic immunotherapy in liver tumors. Further prospective studies incorporating immune profiling are warranted to elucidate the underlying mechanisms and long-term benefits of this approach.

## Introduction

Adrenocortical carcinoma (ACC) is a rare endocrine malignancy with a prevalence of 1–2 cases per million per year (1). It is characterized by an aggressive clinical course and a grim prognosis (1, 2). ACC, once diagnosed, is usually at an advanced stage and with distant metastases, most commonly in the liver, lungs, lymph nodes, and bone (2). Complete surgical resection is the only curative treatment option. However, ACC has a high recurrence rate of 50-70% post-resection, suggesting that patients benefit from adjuvant systemic therapy (3). Adrenolytic therapy with mitotane is a widely used treatment regimen for patients with advanced ACC and is increasingly used also in an adjuvant setting (3). For patients with advanced disease, a more aggressive treatment regimen (mitotane with EDP (etoposide + cisplatin + doxorubicin)) is recommended based on the FIRM-ACT randomized controlled trial (3).

Metastatic ACC carries a dismal 5-year survival rate of less than 15% (4). The liver is a common metastatic site in ACC, and meta-analyses pooling overall survival data demonstrate that liver metastases negatively impact survival regardless of primary malignancy (5). In recent years, locoregional and ablation therapies have been widely used to treat patients and have shown promising outcomes in metastatic and primary liver tumors (6, 7). These ablative therapies can be either thermal or electric-based. Thermal techniques, such as radiofrequency ablation (RFA), microwave ablation (MWA), and laser ablation use heat to destroy tumor cells and unwanted tissue, while cryoablation uses extreme cold (8). However, these ablation approaches are typically not recommended in tumors present at high-risk sites such as the hepatic dome, hepatic capsule, adjacent to at risk organs including colon, stomach and gall bladder, and/or peribiliary region (9). Recently, pulse electric field (PEF) has emerged as a promising alternative to tumors that were previously deemed non-ablative (10). PEF ablation is a recently U.S. Food and Drug Administration (FDA) 510K–cleared electrical ablation modality for soft tissue ablation using biphasic electrical waveforms through a 19-gauge monopolar electrode (11, 12).

Herein, we report an abscopal effect after a pulse electric field intervention in a 49-year-old female with metastatic adrenocortical carcinoma to the liver. Abscopal effect refers to an effect on lesions distant from treated lesions. The patient presented with a history of left salpingo-oophorectomy, hepatic segment 3 wedge resection, and left adrenalectomy. We performed a PEF on 2 of 6 viable tumors and observed a complete response in 3 of the 4 remaining lesions 2- month post-treatment. We posit that the present case report will provide novel insight into the treatment of advanced metastatic liver tumors and the utility of electric-based ablative techniques in clinical practice.

This report aims to highlight the potential abscopal effect of PEF in a patient with metastatic ACC. We present the following case in accordance with the CARE reporting checklist.

## Case presentation

A 49-year-old female patient first presented with a 2- to 3-year history of hypertension, male-patterned hair loss occurring over the past year, and the onset of menopause at age 46. Additionally, she reported a hoarse voice, voice deepening and a 10-pound weight gain that developed over several years.

Evaluations by otorhinolaryngology for voice hoarseness included a chest CT, which revealed mediastinal lymphadenopathy, several pulmonary nodules, nodularity in the anterior mediastinum, a sclerotic lesion in the T5 vertebra measuring 0.9 cm, and a large 8 x 6 cm left-sided adrenal mass that raised concerns for malignancy.

**Figure 1:**
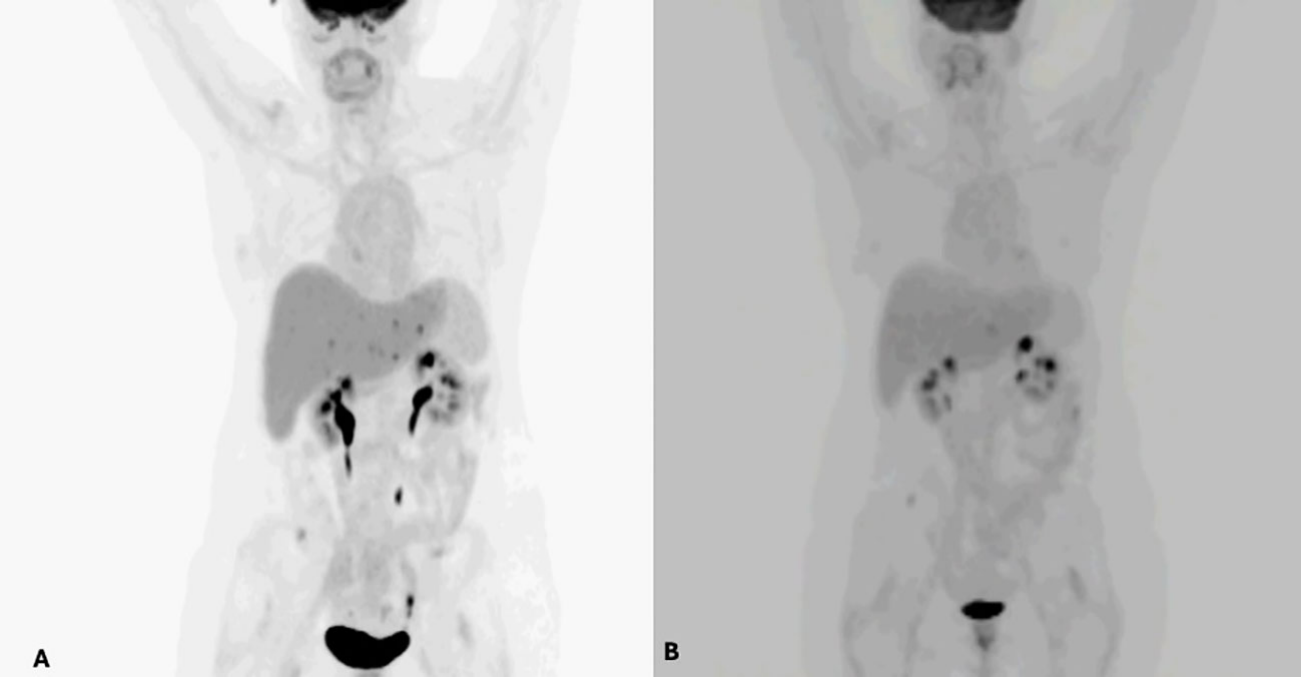
**(A)** PET/CT revealed six scattered hypermetabolic metastatic lesions in the left lobe. **(B)** Post PEF demonstrating resolution of five lesions within left hepatic lobe.

Staging imaging (CT abdomen/pelvis, MRI, and CT chest) demonstrated a large heterogeneous left adrenal mass (9.7 x 8.0 x 12 cm) suspicious for adrenal cortical carcinoma, a loculated left adnexal/ovarian mass (4 x 4 x 7 cm), scattered bilateral pulmonary micronodules with subcentimeter thymic bed nodularity, as well as an inferior hepatic margin 0.8 cm lesion that could be consistent with metastases as well (**Figure 1**).

**Figure 2:**
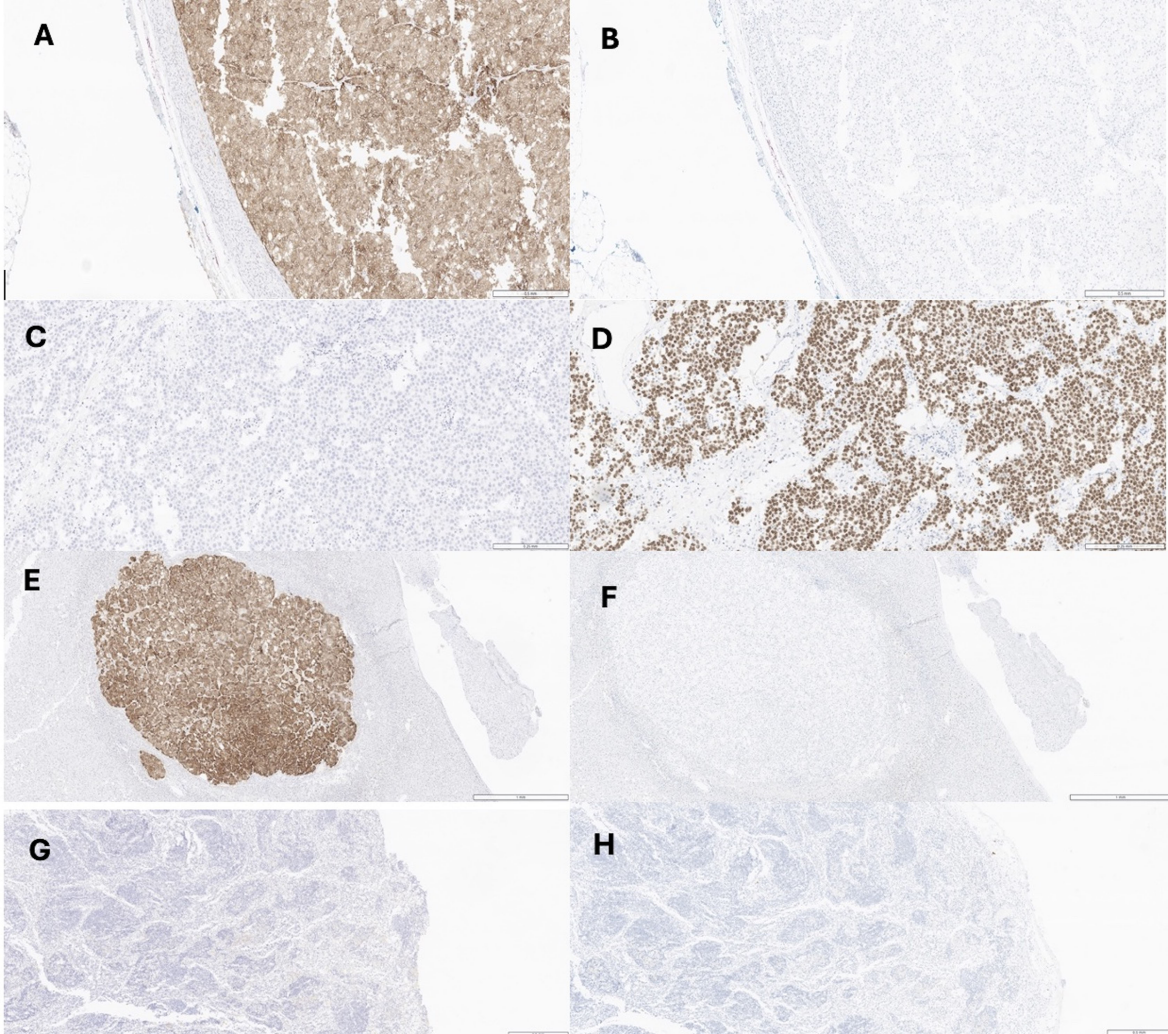
Immunostaining **(A)** Positive immunostaining for α-inhibin in Adrenalcorticalcarcinoma (anti–α-inhibin with hematoxylin counterstain, original magnification 5X). **(B)** Negative immunostaining for SALL4 in Adrenalcorticalcarcinoma(anti–SALL4 with hematoxylin counterstain, original magnification 5X). **(C)** Negative immunostaining for α-inhibin in Dysgerminoma (anti–α-inhibin with hematoxylin counterstain, original magnification 10X). **(D)** Positive immunostaining for SALL4 in Dysgerminoma (anti– SALL4 with hematoxylin counterstain, original magnification 10X). **(E)** Positive immunostaining for α-inhibin in liver (anti–α-inhibin with hematoxylin counterstain, original magnification 1X) **(F)** Negative immunostaining for SALL4 in liver (anti–SALL4 with hematoxylin counterstain, original magnification 1X). **(G)** Negative immunostaining for α-inhibin in lymph node (anti–α-inhibin with hematoxylin counterstain, original magnification 40X). **(H)** Negative immunostaining for SALL4 in lymph node (anti–SALL4 with hematoxylin counterstain, original magnification 40X).

A few weeks later, the patient underwent left salpingo-oophorectomy, hepatic segment 3 wedge resection, and left adrenalectomy. Following the surgery, her cushingoid features abated. Pathology and staging scans confirmed Stage IV (pT3M1) pMMR adrenal cortical carcinoma with liver metastases with cortisol, androstenedione, testosterone secretion. Immunohistochemical stains provided further insight to the origin of each lesion. The ovarian mass demonstrated strong diffuse nuclear expression of SALL4 and OCT4, and strong diffuse membranous CD117 expression, with negative inhibin, supporting a germ cell tumor–type immunophenotype rather than metastatic adrenal cortical carcinoma. Whereas the liver nodule demonstrated diffuse strong inhibin positivity with negative/weak calretinin and negative SALL4, consistent with metastatic adrenal cortical carcinoma. Similarly, the adrenal mass demonstrated diffuse strong inhibin expression, negative/weak calretinin, negative SALL4, weak to moderate membranous β-catenin expression in a subset of cells, patchy/wild-type p53 staining, and a Ki-67 proliferation index of 20%, confirming the primary adrenal cortical carcinoma.(**Figure 2**)

Evaluation of regional lymph nodes (showed negative inhibin, with no evidence of metastatic involvement. Immunostains for mismatch repair proteins (PMS2, MLH1, MSH2, MSH6) on all specimens demonstrated intact nuclear expression, indicating a proficient mismatch repair (pMMR) tumor with a low probability of a microsatellite instability–high (MSI-H) phenotype. (**Figure 2**)

Her family history included a mother with renal cancer and recurrent breast cancer at 65 years old, a father with prostate cancer and pancreatic cancer, and a maternal aunt with lung cancer.

### Oncological and interventional history

When she first presented to oncology, her 9 am cortisol was 39.3 (166–507) nmol/l, serum free testosterone was 164 (0.1–1.7) pg/ml, and dehydroepiandrosterone (DHES) was 702 (0.03–5.8) μg/ml.

Following the surgery, her 9 am cortisol was 286.9 (166–507) nmol/l, serum-free testosterone was 1.1(0.1–1.7) pg/ml, and dehydroepiandrosterone (DHES) was 97 (0.03–5.8) μg/ml.(**Figure 2**)

The patient was started on doxorubicin, etoposide, and cisplatin-mitotane (EDP-M). Unfortunately, after 2 cycles, she had progression with an increase in size and number of multiple arterially enhancing hepatic lesions compared to prior imaging. The patient was then started on second-line treatment with pembrolizumab + mitotane.

Concurrently, the patient was referred to interventional radiology, where she underwent yttrium-90 radioembolization for lesions for progression of lesions in the right hepatic lobe.

Four months post-Y90, PET/CT demonstrated no evidence of viable disease in the right lobe of the liver with 6 scattered hypermetabolic metastatic lesions in the left lobe.

**Figure 3:**
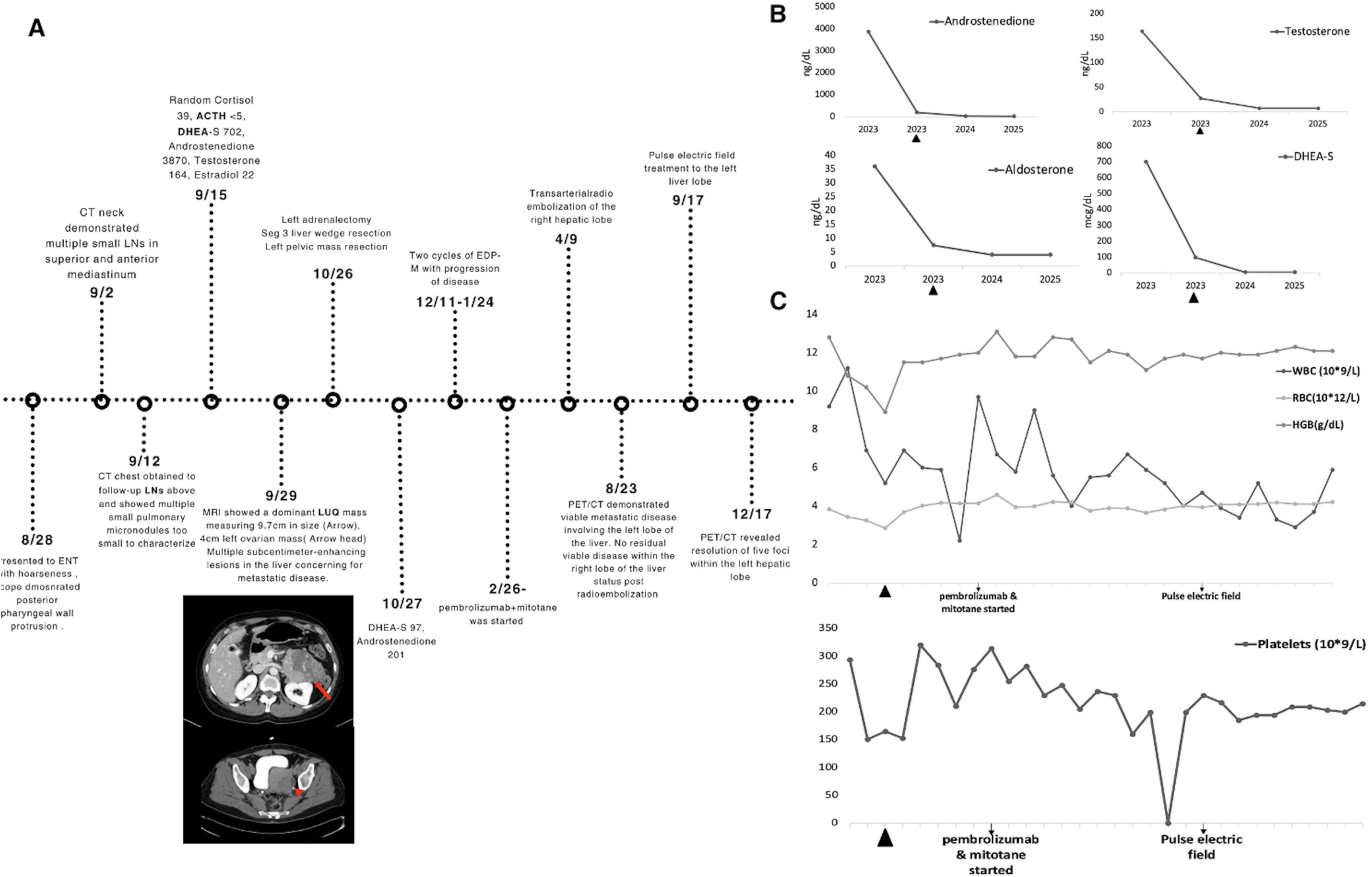
**(A)** Timeline of treatment administration From Episode of care. **(B)** Longitudinal changes in steroid hormone levels following Adrenalectomy **(C)** Longitudinal hematologic trends. ENT, Ear Nose throat; CT, computed tomography; MRI, Magnetic resonance imaging; LN, Lymph node; ACTH, Adrenocorticotropic hormone; DHEAS-S, Dehydroepiandrosterone sulfate; LUQ left upper quadrant; EDP-M, doxorubicin, etoposide, and cisplatin-mitotane; PET, Positron emission tomography scan; WBC, white blood cell; RBC, red blood cell count; Hgb hemoglobin. Arrowhead indicates when adrenalectomy was performed.

Ablation of 2–3 lesions of the left lobe at a time was recommended after a multidisciplinary discussion: an anterior 2.0 cm lesion in hepatic segment 4B and a posterior 1.8 cm lesion in hepatic segment 2 (**Figure 1**). Details of the procedure are discussed below. The timeline is provided in **Figure 3**.

### Procedure details

As detailed above, after a multidisciplinary discussion and shared decision-making with the patient, PEF was chosen to treat low burden viable tumors in the left lobe of the liver in a staged fashion. Although the initial treatment plan was to target all six FDG-avid lesions, only two lesions were treated during the first session due to the procedural time required for pulse-electric-field (PEF) ablation, which remains a significant practical limitation of this technology. A staged approach was therefore selected, with plans to treat the remaining four lesions in a subsequent session. Since the patient was on concurrent immunotherapy and critical location of tumors, 2 of the 6 FDG avid lesions were ablated followed by restaging PET/CT in 2 months.

The procedure was performed under general anesthesia. Using CT guidance, the PEF probes were advanced and positioned within the target(s). For each target lesion, the probes were placed and repositioned as necessary to achieve the desired complete ablation zone. Two probes were placed in each lesion A total of 6 activation cycles were performed. The first lesion in Segment 2, which was 1.8 cm in diameter, received four activation cycles, while the second lesion in Segment 4B, which was 2.0 cm in diameter, received 4 activation cycles as well. (**Figure 3**) At the end of the session, the probes were removed, with no post-procedural adverse effects. The patient continued treatment with pembrolizumab and mitotane, restarted 12 days after her procedure, and continued every 4 weeks. Systemic laboratory trends across her treatment course were also monitored. Notably, following PEF ablation, hemoglobin and platelet counts demonstrated improvement (**Figure 3**).

**Figure 4:**
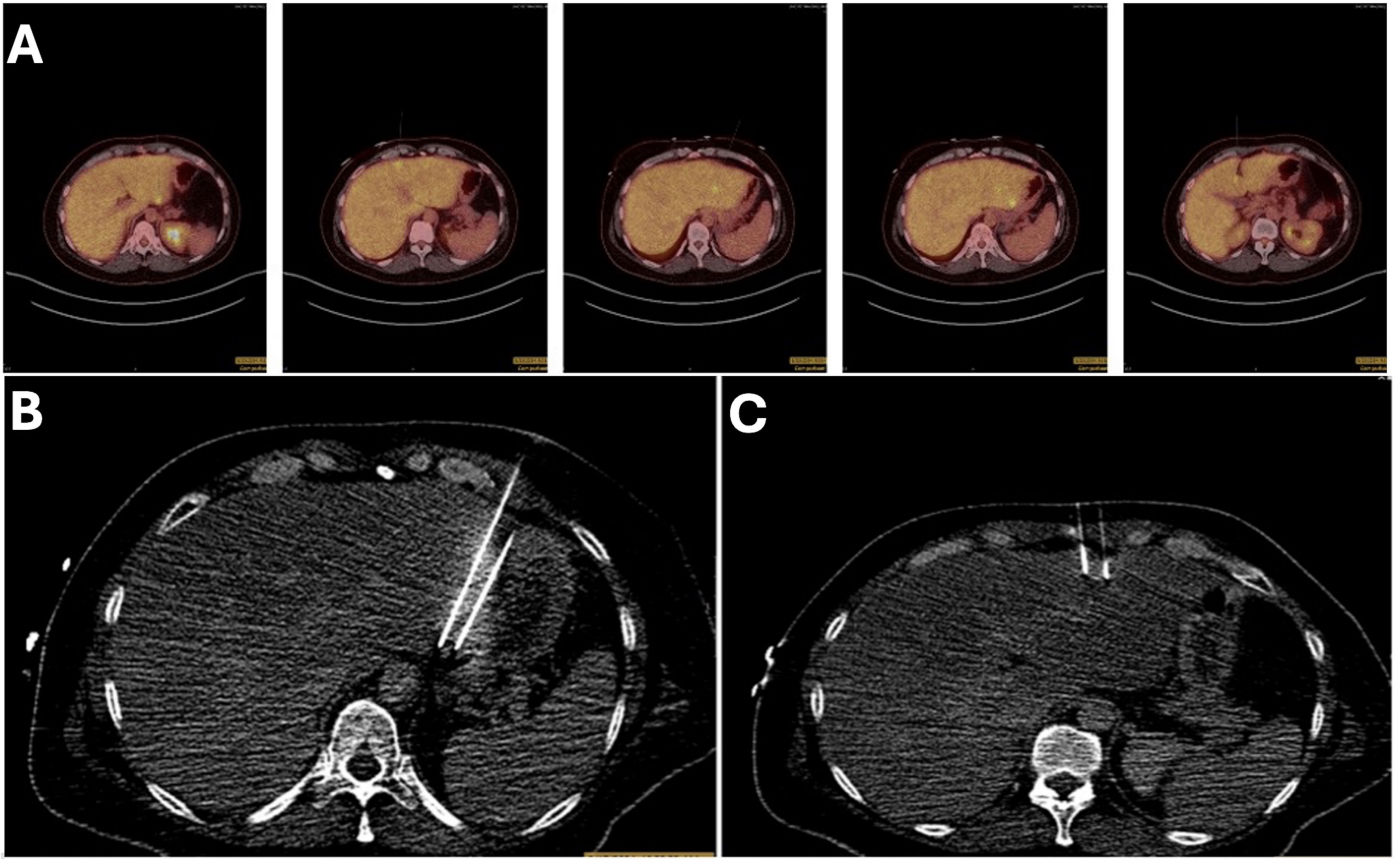
PET/CT scan **(A)** Pre PEF demonstrating 6 FDG avid lesions in the liver. **(B)** post PEF demonstrating resolution of 5 lesions within the left hepatic lobe.

Two months post-PEF ablation, the patient reported satisfaction with her results and the multidisciplinary treatment she has received to date. Her restaging PET/CT revealed resolution of 5 hypermetabolic lesions within the left hepatic lobe, with 1 persistent area of mildly increased uptake and ill-defined hypoattenuation on CT in the anterior aspect of hepatic segment 2/3, which was thought to represent viable disease *vs*. early post-ablation changes (**Figure 4**).

## Discussion

In this report, we present a case of a patient with metastatic liver tumor who demonstrated an abscopal effect after local ablation using PEF. The patient had demonstrated chemotherapy resistance with EDP-M and was later switched to immunotherapy with pembrolizumab. However, her liver disease had remained stable on immunotherapy. After undergoing PEF ablation using the Aliya system (Galvanize Therapeutics, Redwood, CA), we observed radiographic resolution of majority distant metastatic lesions in the liver, suggesting a potential immunomodulatory effect of PEF. Abscopal effect using PEF was recently reported in a patient with vulvar melanoma refractory to dual immune checkpoint inhibitor therapy, demonstrating PEF’s potential in different malignancies (10).

The abscopal phenomenon is exceedingly rare and understudied, first coined by R.H. Mole in 1953 (13, 14). It is derived from Latin word “scopus” meaning “target for shooting at” and “ab” “position away from” (13, 14). The phenomenon describes the partial or complete regression of lesions that were not the target of the treatment (13, 14). Historically, abscopal responses have been reported with radiation therapy in immunogenic tumors such as renal cell carcinoma, melanoma, and lung cancer. However, in less-immunogenic tumors such as breast, HCC and pancreatic cancer, literature is exceedingly rare. Given the rarity of the phenomenon, it’s underlying mechanisms remain poorly understood (15). Adding to this complexity is the lack of heterogeneity of the tumors that exhibit an abscopal effect.

PEF ablation is a recently U.S. FDA 510K–cleared electrical ablation modality for soft tissue ablation using biphasic electrical waveforms through a 19-gauge monopolar electrode (11, 12). The technology disrupts the cell membrane, resulting in osmotic imbalance and cell swelling (16). Unrestricted cell swelling after permeabilization induces necrosis in the targeted cells in a process called irreversible electroporation (IRE), which eventually causes cell death while preserving tumor antigens (8, 16). In theory, PEF then sensitizes the immune system, thus laying the groundwork for systemic therapy to exert its effect.

Preclinical studies suggest that PEF stimulate immunogenic cell death pathways via the release of damage-associated and pathogen-associated molecular patterns (DAMPs and PAMPS, respectively). This stimulates release of immunostimulatory cytokines, which may explain the proposed immunological response observed (8, 17). Furthermore, PEF ablation has been shown to induce the formation of tertiary lymphoid structures (TLSs) in the tumor microenvironment (TME), which are predictive biomarkers for responsiveness to immunotherapy and may contribute to adaptive immune responses against the tumor (18). Thus, PEF may be able to convert a “cold” tumor largely infiltrated by immunosuppressive M2-like tumor-associated macrophages (TAMs) and regulatory T (Treg) cells into a “hot” or a pro-inflammatory state tumor exhibiting infiltration by cytotoxic T lymphocytes (CTLs), natural killer (NK) cells, and enhanced IFN-γ signaling within the TME. This environment further augments the mechanism of action of immune checkpoint inhibitors (17). This proposed model is supported by preclinical studies demonstrating an augmented therapeutic effect of immune checkpoint inhibitors following PEF across various murine cancer models (19, 20).

To the best of our knowledge, this is the first case that showed a durable abscopal effect of PEF in the liver. PEF eliminated two lesions and exerted an abscopal effect on the distant lesions by heightening the immune system. Despite these promising findings, several confounding factors must be considered. The patient received EDP-M and later switched to pembrolizumab, an anti-PD-1 checkpoint inhibitor known to enhance immune responses (12). This could suggest that the abscopal effect observed could be attributed to the systemic therapy itself. Mitotane was also found to potentiate the cytotoxicity of certain chemotherapeutic drugs; however, its ability to potentiate ablative treatment has yet to be established (21). Nevertheless, the patient’s metastatic disease to the liver remained stable while on the combination of pembrolizumab for 7 months prior to PEF. Hence, it remains plausible that PEF may have contributed to an augmented immune response, congruent with previously published preclinical and clinical reports (10, 11, 19).

Currently, evidence supporting the efficacy of PEF in patients with liver tumors remains limited to a few retrospective studies, none of which have reported an abscopal effect (11, 22). Prospective studies incorporating immune profiling, cytokine analysis, and TME characterization are necessary to understand the mechanisms underlying PEF-induced immune modulation in the liver. This case report has several limitations, including a single patient, limited follow-up duration, and lack of immune mediators monitoring in blood to assess antitumoral immune response.

Our findings suggest that PEF ablation may represent a novel strategy for inducing immunogenic cell death in liver tumors. Further prospective studies are warranted to evaluate the long-term efficacy and its role within oncological treatment paradigms.

## Data Availability

The original contributions presented in the study are included in the article/**Supplementary Material**. Further inquiries can be directed to the corresponding author.
